# Assessment of Regeneration Potential in the Clonal Macrophyte *Miscanthus sacchariflorus* (Poaceae) after Burial Disturbance Based on Bud Bank Size and Sprouting Capacity

**DOI:** 10.1371/journal.pone.0120846

**Published:** 2015-03-18

**Authors:** Xinsheng Chen, Chenshu Cao, Zhengmiao Deng, Yonghong Xie, Feng Li, Zhiyong Hou, Xu Li

**Affiliations:** 1 Key Laboratory of Agro-ecological Processes in Subtropical Region, The Chinese Academy of Sciences, Changsha, Hunan, China; 2 Dongting Lake Station for Wetland Ecosystem Research, Institute of Subtropical Agriculture, The Chinese Academy of Sciences, Yueyang, Hunan, China; 3 Department of Garden and Food Processing, Shangqiu Polytechnic, Shangqiu, Henan, China; USDA-ARS, UNITED STATES

## Abstract

The demography of the bud bank and its sprouting capacity are important for understanding the population dynamics of clonal plants and their potential responses to disturbances. To this end, we investigated the size and composition of the bud bank of *Miscanthus sacchariflorus* (Maxim.) Hack. immediately after flooding (November), in winter (January), in spring (March), and before flooding (May) in the wetlands of Dongting Lake. We then examined the sprouting capacity of axillary buds after sediment burial at 0, 5, 10, 15, and 20 cm. Total bud density of *M*. *sacchariflorus* ranged from 2524 buds m^-2^ in November to 4293 buds m^-2^ in March. Rhizome segments with inactive axillary buds, which represented the majority of the bud population (88.7% in November, 93.3% in May), did not sprout during the 140 days of the experiment (n = 250). The sprouting ratio was the highest for active axillary buds buried at 0 cm (64%) and decreased when buried at 10–20 cm (34%–40%). Due to the large number of active axillary buds in the bud bank (211–277 buds m^-2^ from November to the following March), *M*. *sacchariflorus* could completely replace its aboveground shoot population, except in May (142 buds m^-2^). Increasing burial depth delayed bud emergence and reduced the growth period of shoots; however, burial depth did not affect the resulting plant height and only reduced the accumulated biomass at 20 cm. Therefore, the belowground bud bank and its strong sprouting capacity are important factors in the maintenance of local populations and colonization of new habitats for *M*. *sacchariflorus* after burial disturbances. The present methodology, which combined measurements of bud bank demography and sprouting capacity, may reflect the regeneration potential of clonal plants after burial disturbances.

## Introduction

Clonal plants are widespread across all biomes and biogeographical regions, particularly in cold, wet, shaded, and nutrient-poor environments [1]. Most perennial clonal plants possess the capacity of both sexual reproduction through seeds and vegetative propagation through bud banks [1, 2]. However, in perennial-dominated ecosystems, such as grasslands and wetlands, vegetative propagation predominates over sexual reproduction, since seedlings contribute negligibly to population recruitment [3, 4]. Therefore, the bud bank plays a fundamental role in the persistence, structure, and dynamics of local populations in these clonal perennials [5–10].

The crucial role of the bud bank in regeneration after a disturbance has been documented in many clonal plants [11–13]. Bud number fluctuates over the course of a year due to the developmental stages of the parent plants and environmental factors limiting plant growth [5–7, 9]. These seasonal changes in the bud bank make vegetative regeneration sensitive to the timing of disturbance [10, 12]. Therefore, it is important to determine the size of the bud bank at different times for understanding the population dynamics of clonal species and their potential responses to disturbance [12].

Theoretically, every bud in the bud bank has the potential to initiate a new shoot and sustain the population [12, 14], and one of the approaches to assess plant regeneration after disturbance is based on bud counts [12]. However, not all buds are equal in regeneration potential because they differ in size, developmental stage, location, and protection [15]. For example, because they include leaf primordia or prophylls, preformed buds exhibit quicker releafing and resprouting responses upon activation than persistent meristems or adventitious buds [7, 15]. Several studies have estimated the size of the bud bank for the dominant species in a community [7, 9, 10] or for the community as a whole [5, 6]. However, the resprouting capacity of each bud type in these banks has rarely been examined, complicating the accurate estimation of plant regeneration after disturbances.

*Miscanthus sacchariflorus* (Maxim.) Hack. (Poaceae) is a perennial, typical rhizomatous C4 clonal grass, widely distributed in flooded wetlands across the temperate regions of Asia, which are often disturbed by flooding and the accompanying sedimentation [16]. The belowground buds on the rhizomes of this plant are the main reproductive source for population recruitment [14, 17]. After disturbances, such as flooding, buds on rhizome fragments that have been buried in sediment can sprout into shoots [14]. In the present study, we investigated the size, composition, and seasonal dynamics of the bud bank of *M*. *sacchariflorus* in the wetlands of Dongting Lake, where this species usually forms tall, monospecific stands that cover 751 km^2^ (approximately 30%) of the wetland area [18]. We also examined the sprouting capacity for the major bud types of the bank in response to sediment burial, thereby assessing the regeneration potential of *M*. *sacchariflorus* populations after burial disturbances. Specifically, we addressed the following two hypotheses: (1) seasonal variation occurs in the size and composition of the bud bank, and (2) the sprouting capacity from the bud bank and subsequent growth decreases with burial depth.

## Materials and Methods

### Ethics statement

The sampling sites of *M*. *sacchariflorus* did not belong to any farms, national parks, or protected areas, so no permissions or permits were required for collecting plant material. Further, the sites did not contain any endangered or protected species.

### Study sites

Dongting Lake (28^o^30′–30^o^20′N, 111^o^40′–113^o^10′E), the second largest freshwater lake in China, is located in northern Hunan Province and acts as a flood basin for the Yangtze River, adjusting its flow by distributary channels. The wetlands are characterized by large seasonal water level fluctuations (up to 15 m); they are completely flooded from June to October and exposed from November to the following May. Sediment accretes at 1.7 cm annually in the lake basin, but in some sites, this rate reaches 20 cm per year [19]. The mean annual temperature is 16.8°C, with hot summers (June–August, 27.3°C) and cold winters (December–February, 5.8°C) [20]. Annual precipitation is 1382 mm, with more than 60% falling between April and August.

### Study species

The culms of *M*. *sacchariflorus* are slender, erect, and 65–160 cm in height [21], and its rhizomes spread extensively. New ramets sprout in March, after which the plants grow rapidly. The plants flower and fruit from October to November and overwinter from December to February using belowground rhizomes. Vegetative growth through buds formed on horizontal rhizomes in the 0–20-cm soil layer is the primary reproductive strategy [14].

### Field investigation

We used three sections of the lake shoreline where *M*. *sacchariflorus* is extensively distributed as study sites, including Beizhouzi (29°10′31.4″N, 112°47′55.9″E), Chapanzhou (28°54′11.8″N, 112°48′34.6″E), and Tuanzhou (29°20′20.8″N, 112°51′05.4″E). At each site, we established a 1-km transect parallel to the lake shoreline in the middle of species zone. We chose five random points along each transect for destructive shoot and belowground bud sampling. The minimum distance between sampling points was 100 m. We recorded the geographical information of each sampling point using a hand-held GPS (UniStrong Odin Series, China). On each sampling date, we excavated one randomly selected quadrat (50 cm × 50 cm) from each sampling point, providing a total of 15 sampled quadrats per sampling date. In each quadrat, we counted and clipped all ramets within the sampling frame. A preliminary study indicated that most rhizomes of this species are distributed within surface soil to a depth of 15 cm. Using a shovel, we excavated the soil within each frame to a depth of 20 cm to ensure that all the rhizomes were collected. We placed each collected sample in a plastic bag and transported them to the laboratory. We collected samples in early November 2010 (approximately 1 week after flooding), mid-January 2011 (the coldest month), early March 2011 (after the spring sprout), and early May 2011 (before flooding).

### Classification of bud types

We distinguished the bud types of *M*. *sacchariflorus* according to developmental status and their positions on the rhizome [7, 22]. We defined the buds located at the distal ends of young rhizomes as apical buds, while those at the nodes of the rhizomes as axillary buds. We further classified the axillary buds as active or inactive according to their developmental status [22, 23]. We defined axillary buds that contained distinct stem tissue as active axillary buds, while those that had remained quiescent or undeveloped tissue as inactive axillary buds [22]. These active and inactive axillary buds could be differentiated visually based on size, as the active axillary buds were larger (3–20 mm in length) than the inactive axillary buds (usually less than 2 mm in length). Bud bank density was calculated as the number of buds m^-2^.

### Laboratory experiment

We collected the rhizomes from a monodominant stand in Beizhouzi, in the eastern Dongting Lake area (29°10′31.4″N, 112°47′55.9″E) during October 4–6, 2013, approximately 1 week after flooding receded. We excavated rhizomes in 0–20 cm soil layer within a 5-m × 5-m quadrat, stored them in plastic bags, and immediately transported them to the laboratory. We cleaned rhizomes carefully with tap water to protect the integrity of axillary buds and rhizomes. We cut rhizomes into 3–4-cm segments with an axillary bud, either active or inactive, situated centrally.

We planted rhizome segments in five plastic bins (100 cm in length, 100 cm in width, and 80 cm in height) in a greenhouse, where the temperature was controlled at 25 ± 2°C during the day and 17 ± 2°C at night and the light was provided by 400-W SON-T ARGO sodium lamps (Philips Company, UK) at a photon flux density of 600 μmol m^-2^ s^-1^ (PAR) with a 14-h photoperiod. We filled each bin with 10-cm sediment, a mixture of sand and loam (1:1 v/v, containing 16% organic matter, 9.9 μg g^-1^ exchangeable N, 0.65 μg g^-1^ P). We applied a 2 × 5 split plot as a randomized complete block design by using five blocks and divided each bin (block) into five plots (100 cm in length, 20 cm in width, and 80 cm in height) and each plot was randomly assigned to one of the five burial treatments (0 cm, 5 cm, 10 cm, 15 cm, and 20 cm). We chose these depths because in natural populations, rhizomes are mostly located within the upper 20 cm of soil [10, 14]. We further subdivided each plot into two sub-plots, and then randomly and evenly planted two types of buds (active axillary buds and inactive axillary buds) into two rows (10 buds per bud type and one bud type per row). There were 10 subplots in each block (2 bud types × 5 burial depths). We watered these bins every day by using tap water (pH 7.21, containing 4.3 μM NH_4_^+^-N, 16.8 μM NO^3^-N, 1.9 μM PO_4_^3^-P), to maintain moist conditions for the substrate.

We recorded shoot emergence daily. The bud was considered as sprouting when a new shoot was higher than 0.5 cm [14]. We concluded the experiment after 140 days because no shoot had sprouted after 130 days. At harvest, we measured the height of the three earliest emerging plants in each subplot, as well as the accumulated biomass of these plants after drying at 80°C for 48 h in an oven.

### Statistical analysis

We evaluated the differences in bud densities, and the proportion of each type of bud to the whole bud population between sampling periods by using linear mixed models, with season as the main factor and sample site as a random factor [24]. Since no inactive axillary buds sprouted, we only analyzed the effect of burial depth on active axillary buds. We calculated the sprouting ratio, emergence time, plant height, and accumulated biomass among different burial depths using a linear mixed model. Multiple comparisons were performed using a two-way analysis of variance in conjunction with Fisher’s least-significant difference (LSD) test and Tukey’s honest significant difference (HSD) test at p < 0.05. All statistical analyses were performed using SPSS v15.0 (IBM, USA).

## Results

### Seasonal patterns of bud bank density and shoot density

**Fig 1:**
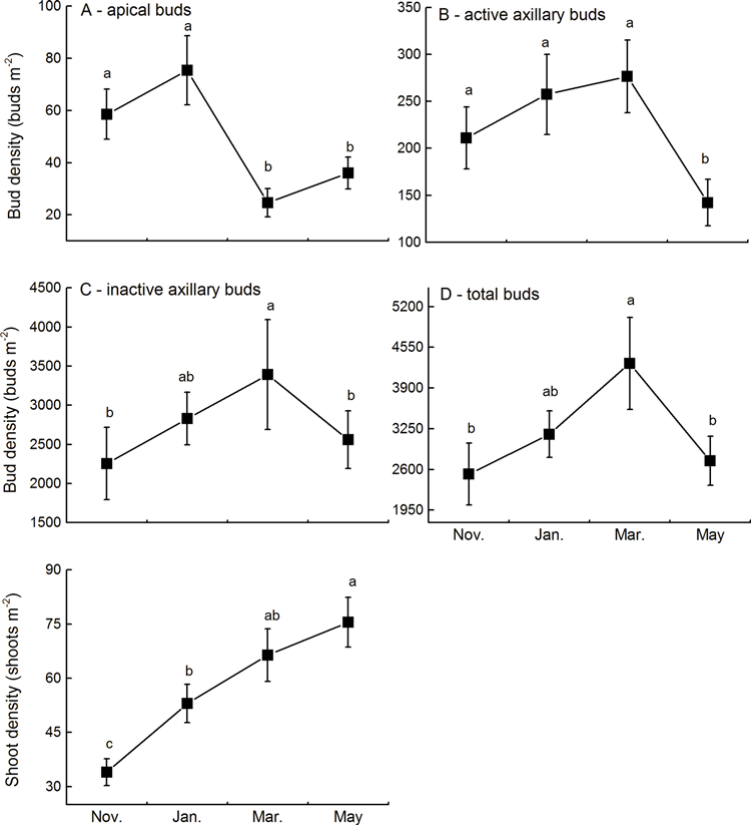
Density changes in apical buds (A), active axillary buds (B), inactive axillary buds (C), total buds (D), and shoots (E) of *Miscanthus sacchariflorus* from November 2010 to May 2011 in the wetlands of Dongting Lake. Data are expressed as mean ± standard error (SE). Different lowercase letters indicate significant differences between seasons at *P* < 0.05.

The density of apical buds collected in January (75 ± 13 buds m^-2^) was significantly higher than that of apical buds collected in March (25 ± 5 buds m^-2^) (Fig. 1A). The density of active axillary buds did not show any significant change from November through the following March (from 211 ± 33 buds m^-2^ to 277 ± 39 buds m^-2^) and only decreased significantly in May (142 ± 25 buds m^-2^) (Fig. 1B). The density of inactive axillary buds increased continually from November through March (from 2254 ± 462 buds m^-2^ to 3391 ± 702 buds m^-2^) and then significantly decreased in May (2559 ± 368 buds m^-2^) (Fig. 1C). The seasonal pattern of total rhizome buds was similar to that of inactive axillary buds (Fig. 1D). The density of shoots increased continually from November through May (from 34 ± 4 shoots m^-2^ to 76 ± 7 shoots m^-2^) (Fig. 1E).

### Seasonal composition patterns of all bud types

**Fig 2:**
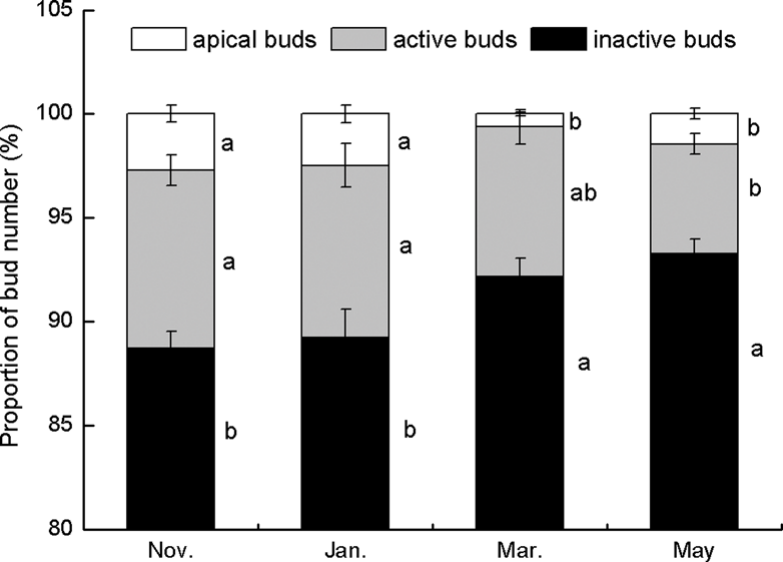
Percentages of apical buds, active axillary buds, and inactive axillary buds collected between November 2010 and May 2011. Data are expressed as mean ± standard error (SE). Different lowercase letters indicate significant differences between seasons at *P* < 0.05.

Inactive axillary buds represented the majority of the bud population throughout the growing season (88.7% in November, 93.3% in May); however, the ratio of each bud type to total bud density varied significantly between the sampling periods (*P* < 0.05, Fig. 2). The proportion of apical buds to total bud density decreased from January (2.5%) to March (0.6%) (Fig. 2), while the proportion of active axillary buds to total buds decreased from January (8.3%) to May (5.3%). Conversely, the proportion of inactive axillary buds to total buds increased from January (89.2%) to March (92.2%) (Fig. 2).

### Sprouting ratio and emergence time

**Fig 3:**
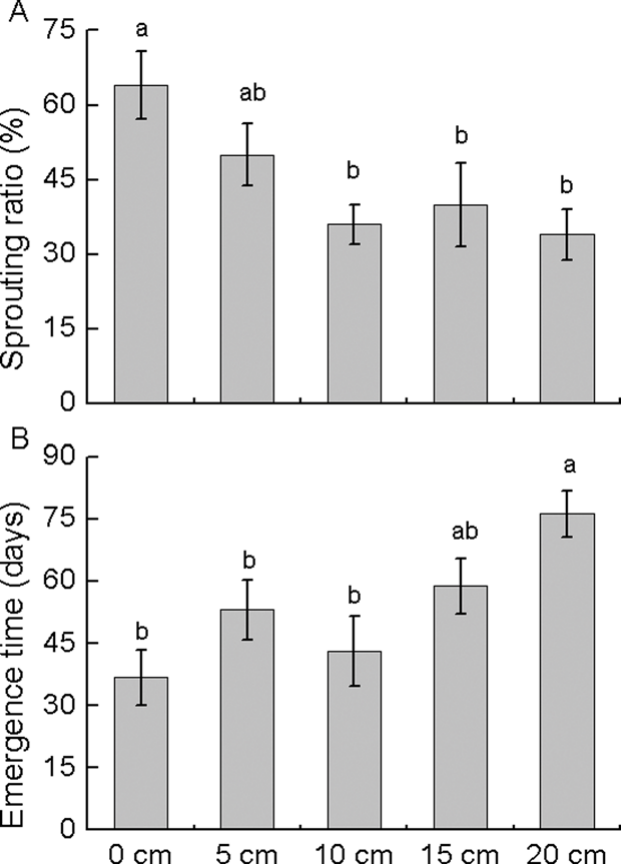
Sprouting ratio (A) and emergence time (B) of active axillary buds at different burial depths. Data are expressed as mean ± standard error (SE). Different lowercase letters indicate significant differences between burial depths at *P* < 0.05.

During the 140 days of the experiment, no rhizome segments with inactive axillary buds sprouted (n = 250). Sprouting was observed in 44.8% of the rhizome segments with active axillary buds, but the sprouting ratio differed among different burial depths (*P* < 0.05, Fig. 3A). The sprouting ratio was the highest for active axillary buds buried at 0 cm (64%) and was significantly lower for buds buried at 10–20 cm (34%–40%). The active axillary buds sprouted 6 days after burial, and no new active axillary buds sprouted at 129 days after burial. The emergence time was the shortest for active axillary buds buried at 0 cm (36.8 days) and was significantly higher for those buried at 20 cm (76.3 days) (*P* < 0.05, Fig. 3B).

### Plant height and accumulated biomass

**Fig 4:**
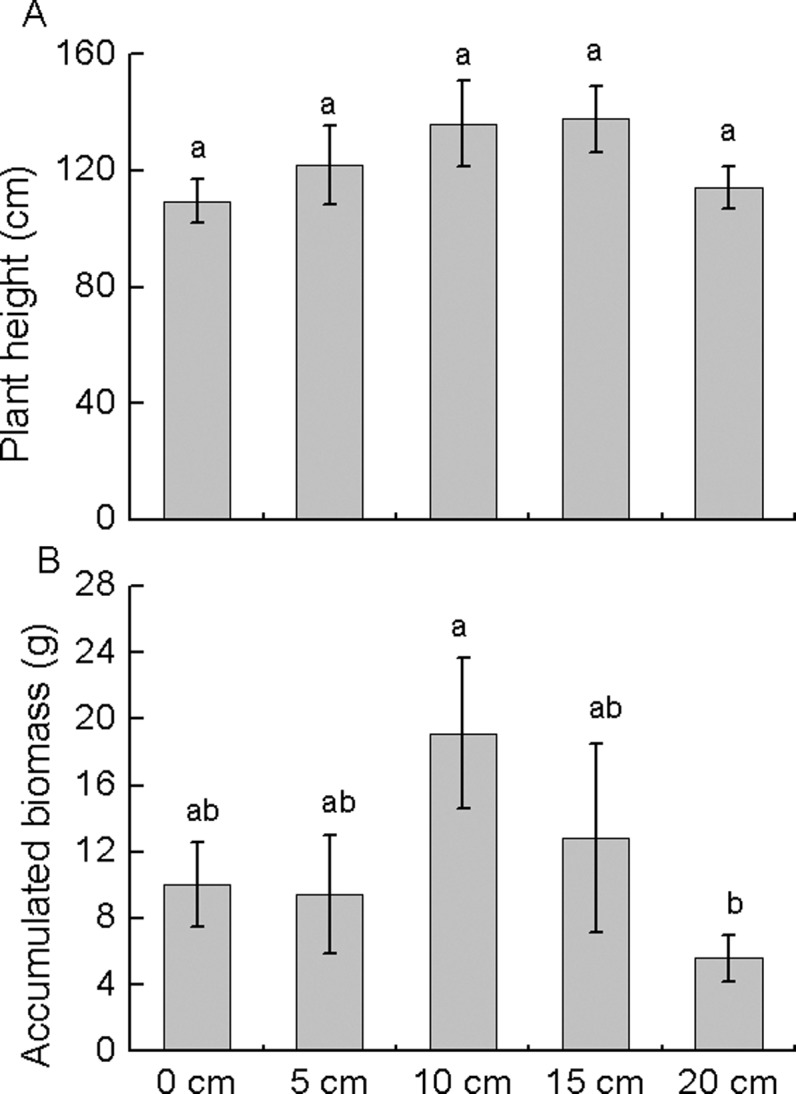
Plant height (A) and accumulated biomass (B) of plants regenerated from active axillary buds at different burial depths. Data are expressed as mean ± standard error (SE). Different lowercase letters indicate significant differences between burial depths at *P* < 0.05.

The height of plants developed from the active axillary buds did not change significantly with burial depth (*P* > 0.05, Fig. 4A). Accumulated biomass was the highest for plants derived from active axillary buds buried at 10 cm (19.1 g), but this value was not significantly different from those buried at 0, 5, or 15 cm (9.5–12.8 g). Biomass was significantly lower for plants derived from buds buried at 20 cm (5.61 g).

## Discussion

### Seasonal dynamics of the bud bank

The density and composition of the bud bank varied among seasons, and the seasonal dynamics of the buds varied among different bud types. These results were consistent with our first hypothesis, which predicted possible seasonal variation in the size and composition of the bud bank. Since all the aboveground shoots of *M*. *sacchariflorus* die over the winter, all new shoots in the spring must sprout from the belowground bud bank. In undisturbed habitats, actively growing apical buds prevent the growth of axillary and adventitious buds situated below the apical meristem [7, 12, 25]. As observed in the present study, the apical buds first sprouted in the spring and consequently decreased significantly in density (Fig. 1A). After the apical buds sprout, *i*.*e*., when apical dominance is broken, axillary buds are able to sprout [26]. This pattern may explain why the point at which the active axillary buds decreased (in May) lagged behind the decrease in apical buds (in March) (Fig. 1A, 1B). The density of inactive buds may increase with the growth of new rhizomes and decrease through the transition into active buds or mortality [7, 8, 23].

The density of apical buds of *M*. *sacchariflorus* decreased markedly after spring sprouting, whereas that of axillary buds, both active and inactive, did not decrease significantly after spring sprouting (Fig. 1A-D). In *M*. *sacchariflorus*, it is likely that only a small proportion of buds (mostly apical buds) recruit the shoot population in the spring, and therefore the majority of axillary buds remain dormant even after spring sprouting [6, 10]. The density of shoots of *M*. *sacchariflorus* continues to increase over time, indicating that some buds sprout to recruit shoot population during the growing season. *M*. *sacchariflorus* may make gradual and continual deposits to the bud bank after shoot emergence, perhaps creating a continuously available propagule pool [10].

Notably, *M*. *sacchariflorus* maintained a small bud population, particularly of apical and active axillary buds, before the flooding season (Fig. 1A-D). In the wetlands of Dongting Lake, emergent macrophytes, such as *M*. *sacchariflorus*, experience a long flooding season, normally lasting 4–5 months [27]. The maintenance of a large population of active meristems through this long flooding season may be prohibitively costly in terms of the use of stored carbohydrates [10, 28–30]. Therefore, the maintenance of a small bud population and a low proportion of active meristems before flooding may help the species endure the long flooding season. After floodwaters recede at the end of growing season, *M*. *sacchariflorus* may allocate more energy, such as carbohydrates, to belowground bud banks to ensure the aboveground population recruitment in spring [7, 9, 10].

### Sprouting and plant growth

The majority, if not the entirety, of *M*. *sacchariflorus* resprouting occurs from active axillary buds. A previous study indicated that both meristems and reserves are required for resprouting [15]. Active axillary buds of *M*. *sacchariflorus* are larger than inactive axillary buds, as they include distinct stem tissue. Larger buds represent relatively larger carbon reserves, which promote rapid resprouting upon activation [14]. In contrast, the small upfront investment in the construction and maintenance of inactive buds results in high activation costs, and the small carbon reserves in these inactive buds retard the rate of resprouting [15]. The majority of post-disturbance sprouting in temperate trees occurs from active buds [31], which is consistent with the results of our study.

The sprouting ratio of the active axillary buds of *M*. *sacchariflorus* was decreased after burial at 10 cm or deeper. Sediment burial is thought to be a major environmental stress reducing the emergence and survival of rhizome and stolon fragments [32–34]. After sediment burial, the energy required for resprouting must be derived entirely from resources stored in the buds and rhizome fragments until the new ramet recovers the photosynthetic capacity to support respiration and growth [32, 35, 36]. Beyond a certain burial depth threshold, new sprouts may fail to emerge because the carbohydrate reserves in the storage organs become completely depleted [37]. Nevertheless, 34% of the *M*. *sacchariflorus* rhizome fragments with active axillary buds could sprout even when buried at 20 cm. The strong sprouting capacity of *M*. *sacchariflorus* may be attributed to the high soluble sugar content in its buds and the large biomass accumulated in its rhizomes [14, 38].

Our study suggested that increasing burial depth delayed bud emergence and reduced the growth period of shoots; however, burial depth did not affect the resulting plant height and only reduced the accumulated biomass at 20 cm. The accumulated biomass was actually higher for plants regenerated from buds buried at 10 cm than at shallower depths. These results only partially supported our second hypothesis, which predicted that plant growth possibly decreases with burial depth. Previous studies have suggested that burial may stimulate fragment growth in some cases [32–34, 39]. Enhanced plant vigor following burial may be attributed to increased soil volume, soil resources, activity of mycorrhizal fungi, or a reactive response by the plant [32]. Plants regenerated from buds buried deep in the sediment exhibited similar or even greater performance compared to those regenerated from shallower buds, suggesting that *M*. *sacchariflorus* can recover from severe burial disturbances.

### Regeneration potential from the bud bank after burial disturbance

*M*. *sacchariflorus* has a consistently large bud bank, with total bud density ranging from 2524 buds m^-2^ in November to 4293 buds m^-2^ in March. Although inactive axillary buds, which constituted 88.7–93.3% of the bud bank, could not sprout, more than 34% of the active axillary buds exhibited sprouting. Due to the large number of active axillary buds in the bud bank (211–277 buds m^-2^ from November to the following March), *M*. *sacchariflorus* could completely replace the aboveground shoot population, except in May (142 buds m^-2^). Therefore, the large bud bank and strong sprouting capacity of *M*. *sacchariflorus* are important factors in the maintenance of local populations and colonization of new habitats after disturbances such as flood scouring and sediment burial. We assumed that the resprouting capacity from the active axillary buds of *M*. *sacchariflorus* was the same throughout the growing season. However, the time of year may be a crucial factor, as the nutrient content of rhizomes varies within the growing season in other species [40, 41]. Determining whether this seasonal variation in sprouting capacity exists in *M*. *sacchariflorus* requires further investigation. The present methodology, which combined measurements of bud bank demography and the sprouting capacity of each bud type in these banks, may reflect the regeneration potential of clonal plants after burial disturbances.
